# Description of a new species of *Apterotoxitiades* Adlbauer, 2008 (Cerambycidae, Dorcasominae, Apatophyseini) and the female of *A. vivesi* Adlbauer, 2008, with notes on the biology of the genus

**DOI:** 10.3897/zookeys.482.8901

**Published:** 2015-02-11

**Authors:** Karl Adlbauer, Anders Bjørnstad, Renzo Perissinotto

**Affiliations:** 1 Kasernstraße 84, A-8041 Graz, Austria; 2 Høyåsstien 12, NO-3727 Skien, Norway; 3 School of Life Sciences, University of KwaZulu-Natal, P. Bag X54001, Durban 4001, South Africa

**Keywords:** Cerambycidae, Dorcasominae, Apatophyseini, *Apterotoxitiades*, new species, habitat, South Africa

## Abstract

Following the description of the Apatophyseini genus *Apterotoxitiades* Adlbauer, 2008 (Cerambycidae: Dorcasominae) from South Africa, a new species has now been discovered in the eastern Drakensberg range of the country. The holotype female is here described as *Apterotoxitiades aspinosus* Björnstad, **sp. n.** Also, a new small series collected at Hogsback, in the Amathole range, has allowed the description of the previously unknown female of the type species, *Apterotoxitiades vivesi* Adlbauer, 2008. Both species are high altitude dwellers, occurring above 1300 m asl and their habitat consists mainly of mountain grassland interspersed with mistbelt forest pockets. All specimens were recorded in the austral winter to early spring, when these mountain ranges are occasionally covered in snow and night temperature plummet below 0 °C. They appear to be nocturnal and their complete lack of wings indicates a remarkable adaptation to cold conditions at high altitude.

## Introduction

The genus *Apterotoxitiades* Adlbauer, 2008 was described on the basis of a single male from Hogsback in the Amathole mountains of the Eastern Cape Province of South Africa. It was collected in August 1992, under a large log on gently sloping grassland terrain. In early September 2014, the type locality was revisited, resulting in several new specimens including the hitherto unknown female of the only described species, *Apterotoxitiades vivesi* Adlbauer, 2008. This survey also provided further details on the habitat characteristics and ecology of this species.

A female specimen of what is obviously a representative of the genus *Apterotoxitiades* has for some time been in the ABPC collection. It had been collected in the Drakensberg mountains of KwaZulu-Natal in October 1972, by an unknown collector. Because of a lack of knowledge of the degree of sexual dimorphism within the genus prior to the new Amathole collection, it was not possible to conclude with confidence as to whether this was the unknown female of *Apterotoxitiades vivesi* or a different species. The new material from Hogsback however, clearly shows that the Drakensberg specimen represents an entirely different species which is hereby described.

A brief outline of the generic diagnostic characters is given below. The generic description of *Apterotoxitiades* was provided by Adlbauer (2008), but the discovery of the new species, *Apterotoxitiades aspinosus* sp. n. necessitates a slight amendment of the original description, mainly in virtue of a total absence of lateral pronotal spines in the new species.

## Methods

Specimen length was measured from the anterior margin of the head to the elytral apex. Specimen width represents the maximum width of the elytra. Photos of set specimens were taken using a Canon Eos 5D camera fitted with a Canon MP-E 65 Macro 2.8-1.5× objective. Components of male genitalia were photographed under a Nikon SMZ 25 stereomicroscope, using a Nikon Digital Sight DS-Fi2 camera. *In situ* photos were taken using a Ricoh CX1 camera with macro setting.

Collections are abbreviated as follows: TMSA, Ditsong National Museum of Natural History (formerly Transvaal Museum), Pretoria, South Africa; ISAM, Iziko South African Museum, Cape Town, South Africa; NHMO, Natural History Museum, Oslo, Norway; ABPC, Anders Bjørnstad Private Collection, Skien, Norway; KAPC, Karl Adlbauer Private Collection, Graz, Austria; RPPC, Renzo Perissinotto & Lynette Clennell Private Collection, Port Elizabeth, South Africa. Geographical abbreviations are as follows: RSA, Republic of South Africa; KZN, KwaZulu-Natal Province, South Africa; EC, Eastern Cape Province, South Africa.

## Taxonomic account

### Genus *Apterotoxitiades* Adlbauer, 2008

The major characters are the wingless body with strongly atrophic (nearly absent) shoulders, short head with small, coarsely facetted eyes and long palpi with terminal segment expanded in male. Pronotum armed or not (amended from the original description). Legs are long and slender, coxae are rather large and prominent. In the light of the recent work undertaken by Villiers et al. (2011), on the Dorcasominae of Madagascar, it is clear that the male genitalia of *Apterotoxitiades* fall within the range described for this subfamily.

Only a single male of the remarkable type species *Apterotoxitiades vivesi*, which seemingly occurs rare and local, was hitherto known from the genus. The new material recently found allows the description of the female.

#### Apterotoxitiades vivesi

Taxon classificationAnimaliaColeopteraCerambycidae

Adlbauer, 2008

**Figure 1.**
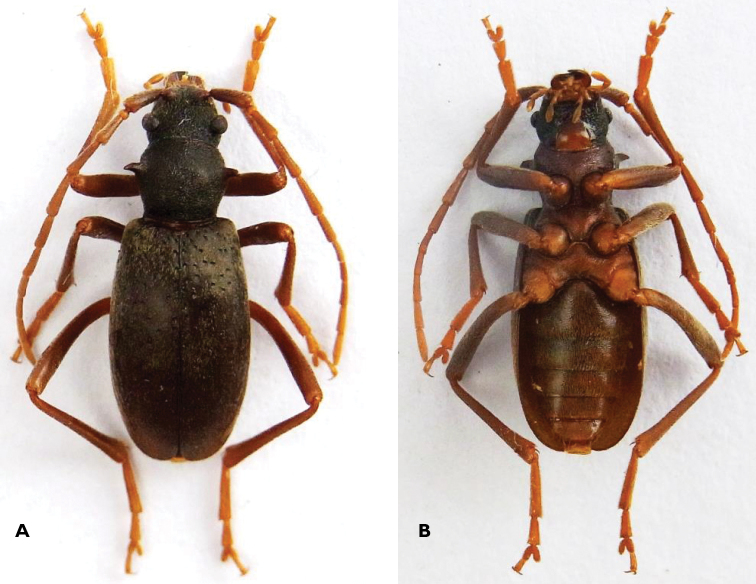
*Apterotoxitiades vivesi*: Female dorsal (**A**) and ventral (**B**) habitus, 11 mm TL (Photos: Lynette Clennell).

**Figure 2.**
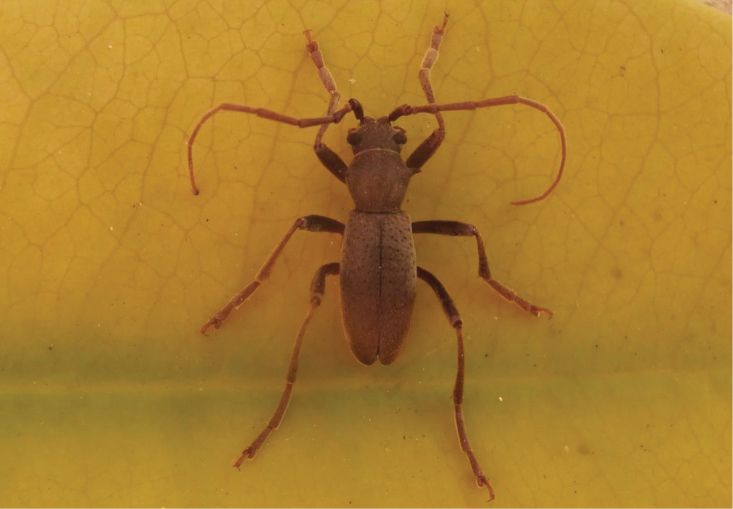
*Apterotoxitiades vivesi*: Male specimen in its natural habitat, Hogsback Forestry, September 2014 (Photo: Lynette Clennell).

**Figure 3.**
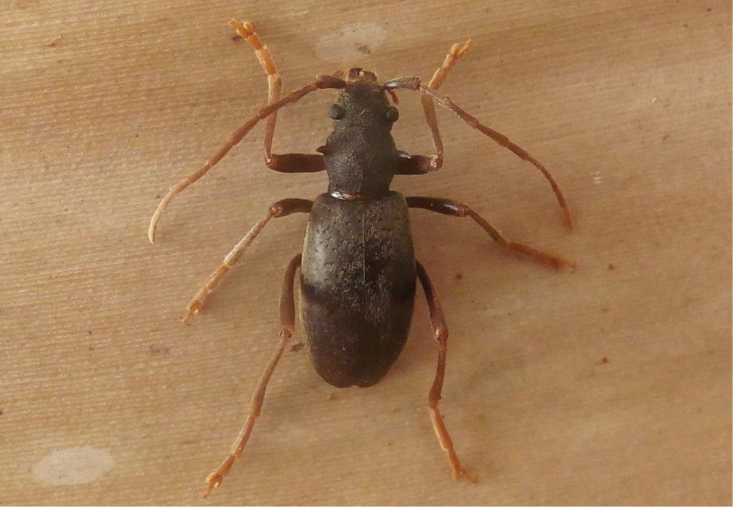
*Apterotoxitiades vivesi*: Female specimen in its natural habitat, Hogsback Forestry, September 2014 (Photo: Lynette Clennell).

**Figure 4.**
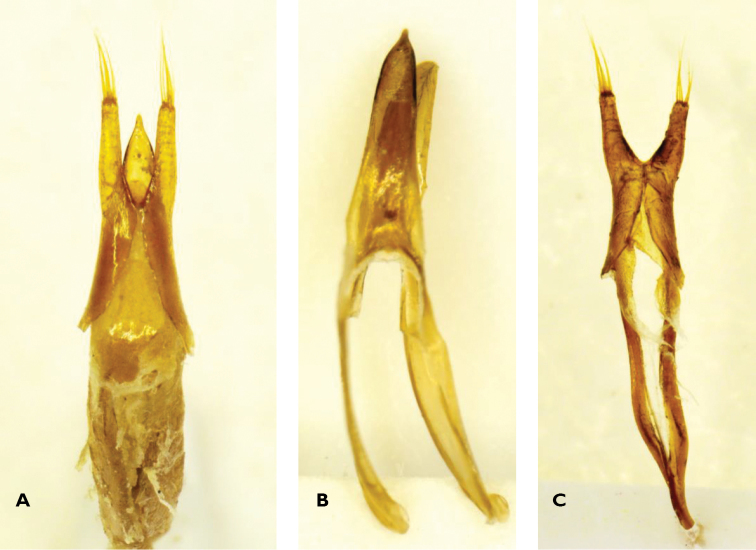
*Apterotoxitiades vivesi*: Dorsal view of whole male genitalia (**A**), aedeagus (**B**) and tegmen (**C**) (Photos: Lynette Clennell).

Figures 1 , 2 , 3 , 4

##### Material examined.

Four female and two male specimens with data: South Africa, EC, Hogsback, 1300 m, 7 Sep 2014, R. Perissinotto & L. Clennell leg. (TMSA, ISAM, KAPC, RPPC). Only one male and one female were found still alive, while the other four specimens were already dead, two with soft tissue consumed by spiders.

##### Description.

♀. Length: 10–11.5 mm; width: 3.5–4 mm (n = 4). General habitus as in male (Figure 2), but with shorter antennae and legs and wider elytra (Figures 1–3).

*Coloration*. Dark greyish brown, apices of the elytra slightly lighter brown. Palpi, antennae, legs and ventral side light yellow brown. Mandibles light yellow brown, with the exception of the apices which are black.

*Body surface*. Whole surface covered in short depressed silky tomentum. Long, thin, hairlike whitish grey bristles present especially on the lateral side of the mandibles, scapus and pronotal sides (Figure 1A).

*Head*. Broad with strong, falciform mandibles. Palpi moderately long, terminal segment only very weakly enlarged. Eyes coarsely facetted, strongly protuberant and broadly separated, small, oblique, not emarginate and far behind antennal tubercles. Frons between the eyes broad and flat. Antennae reaching to the second half of the elytra. Antennomeres becoming shorter towards the end, but not very different in length from each other.

*Pronotum*. As long as wide with long, rather acute lateral spines pointed strongly obliquely upwards (Figure 1A). Surface like in male (Figure 2). Disc convex in the middle. Unlike in male, the anterior edge is not broader than the posterior.

*Scutellum*. Very small, hardly visible, wider than long.

*Elytra*. Fused, somewhat broader than in male, widest in the anterior third. Strongly convex, both laterally and dorsally. Slightly over half of the anterior part sparsely punctate. Apices broadly rounded.

*Legs*. Long and slender, but shorter than in male (Figures 2 and 3). Coxae large and projecting (Figure 1B).

*Ventral surface*. All coxae well separated from each other, especially the metacoxae. The first visible abdominal sternite is the longest, with the following becoming progressively shorter until the fifth visible (Figure 1B).

*Male*. A general description is provided in Adlbauer (2008). Only further details of the genitalia, along with photos of whole genitalia as well as tegmen and aedeagus separately are provided here (Figure 4A–C). Aedeagus with heavily sclerotized acute dorsal lobe bearing an acuminate apex. Ventral lobe with a rounded, weakly truncate apex, much shorter than dorsal lobe, and decidedly less sclerotized. Apophyses long, strap-shaped and constituting more than 50% of total aedeagus length (Figure 4B). Tegmen with relatively long and slender, slightly diverging parameres with apical brushes of very long setae (Figures 4A, C). Presence of sheath-like appendage between the base of the parameres and the “tegmen ring”, on both sides.When the aedeagus is in its position inside the tegmen, the apex of the dorsal lobe reaches almost to the apices of the parameres, while the ventral lobe reaches just beyond the point of diversion of the parameres (Figure 4A).

#### Apterotoxitiades aspinosus

Taxon classificationAnimaliaColeopteraCerambycidae

Björnstad sp. n.

http://zoobank.org/704D5D2E-5099-43E7-9478-5EBE9EC9B30A

Figure 5

##### Type.

Holotype (HT) ♀: RSA, Natal 1500/2000 m [Royal] Nat[al] Nat. Park X/1972 [collector unknown] (NHMO).

##### Diagnosis.

The most obvious difference from *Apterotoxitiades vivesi* is the total lack of lateral spines on the pronotum. Both sexes of *Apterotoxitiades vivesi* have pronotum with “langen, zahnförmigen Seitendornen” (Adlbauer 2008). The new species also differs by its greater size (17 mm vs. 10–11 mm in *Apterotoxitiades vivesi* female), and by the somewhat more elongate body outline.

##### Etymology.

The word “*aspinosus*” refers to the lack of lateral spines on the pronotum, which are on the other hand very prominent in the type species, *Apterotoxitiades vivesi*.

##### Description.

**Figure 5.**
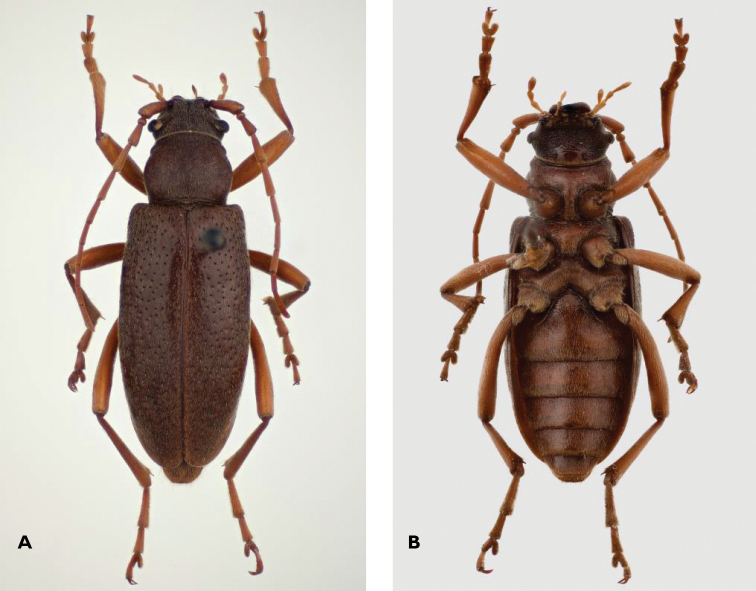
*Apterotoxitiades aspinosus* sp. n.: Holotype female dorsal (**A**) and ventral (**B**) habitus, 17 mm TL (Photos: Karsten Sund and Hallvard Elven).

**HT** ♀. Length: 17 mm; width 5.8 mm. Habitus rather slender, long legged, flightless with fused elytra (Figure 5).

*Coloration*. Head and pronotum dark reddish brown, elytra slightly lighter. Legs, antennae and palpi yellow to brownish yellow. Eyes black with bronze lustre.

*Body surface*. Head and pronotum finely, but densely punctate/granulate. Elytra with scattered, shallow pit-like punctation, each pit bearing a pale yellowish bristle. Elytra surface with short, curved ± adpressed silky tomentum. The same type of tomentum occurs on palpi, head, scape and pronotum, but there with interspersed long, stiffly erect pale yellowish-hyaline bristles, particularly distinct on anterior part of head and lateral part of pronotum.

*Head*. Both labial and maxillary palpi long and slender and with ultimate joints narrowly triangular. Mandibles strong, sickle-shaped with curved, glabrous and shiny apices. Front of head with moderately raised antennal tubercles, and without a longitudinal furrow between them. Eyes small, strongly protuberant, far apart from antennal socket, only sligthly emarginate. Antennae reaching elytral midlength; scapus widened apically; pedicellus almost globular, but shorter than wide. Antennomere 5 of same length as scape, following antennomeres shorter than these and gradually tapering and shortening distally; antennomeres 5–11 with minute, but dense greyish tomentum.

*Pronotum*. Shorter than wide (length/width ratio = 0.8) and with posterior margin wider than anterior. Both edges are only weakly thickened or rimmed. Small constriction on anterior end, at about one fifth of the length, otherwise smoothly convex both dorsally and laterally.

*Scutellum*. Short, broadly triangular with a broad, slightly thickened black border.

*Elytra*. Fused, strongly convex both laterally and dorsally and with evenly rounded apices. Shoulders only weakly marked.

*Legs*. Long and slender with only weakly thickened femora; straight tibiae gradually widening apically; tarsi long and slender, especially the metatarsi.

*Ventral surface*. Gula glabrous, all other parts finely granulate and rather densely covered in curved, silky, adpressed tomentum as on dorsal side (Fig. 5B). Procoxae strong and conical, separated by a narrow prosternal process slightly widened and truncate at apex. Procoxal cavities more or less circular in outline but antero-laterally with a small and short acute extension. Metasternum narrow with a truncated triangular process (Fig. 5B). Visible abdominal sternites 1–5 with a finely granulate microstructure and progressively narrowing posteriorly. Sternite 5 with a straight to weakly concave truncation apically.

*Male*. Unknown.

### Biology of the genus *Apterotoxitiades*

**Figure 6.**
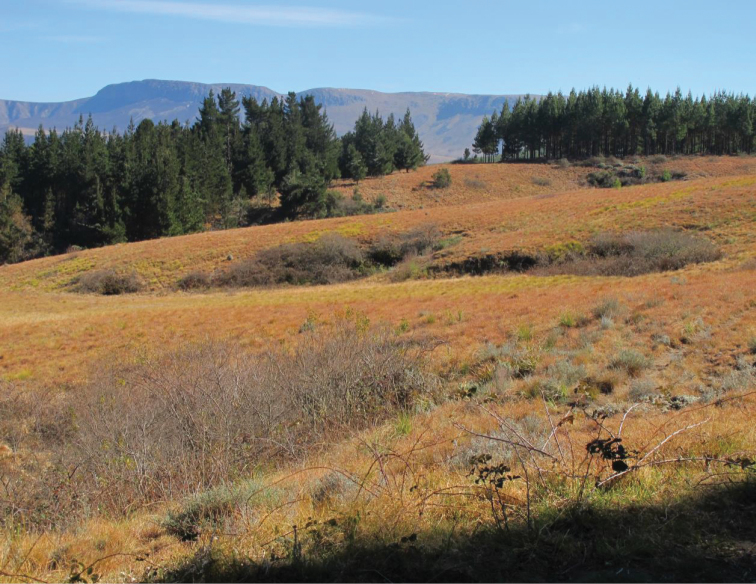
*Apterotoxitiades vivesi*: Typical habitat of mountain grassland with shrub pockets and pine plantations on the slopes of the Hogsback mountain range (Photo: Lynette Clennell).

**Figure 7.**
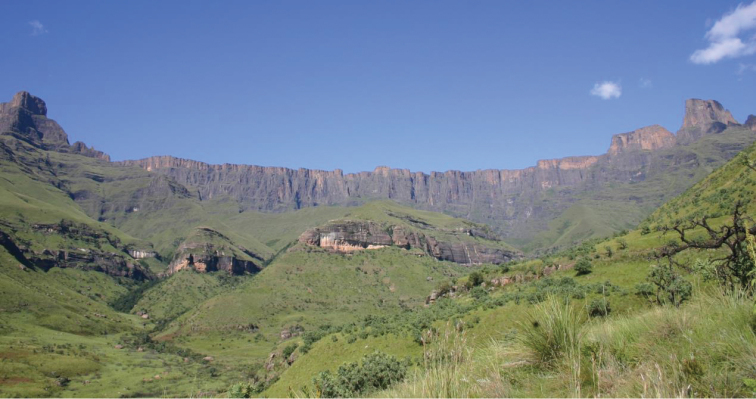
*Apterotoxitiades aspinosus*: The Amphitheatre of the Royal Natal National Park, as a likely representative of the typical Drakensberg habitat of this species (Photo: Gerald Camp).

Both *Apterotoxitiades* species currently known have been collected in grassland terrain at high altitude, above 1300 m asl, in the Amathole range of the Eastern Cape (Figure 6) and the eastern Drakensberg of KwaZulu-Natal (Figure 7). The vegetation units that characterize these areas are typically Amathole Montane Grassland (Gd 1, habitat of *Apterotoxitiades vivesi*) and Northern Drakensberg Highland Grassland (Gd 5, presumed habitat of *Apterotoxitiades aspinosus* sp. n.). Both are part of the Drakensberg Grassland Bioregion (Mucina and Rutherford 2006). The Amathole Montane Grassland unit exhibits short grassland dominated by a variety of grass species, mainly *Themeda triandra*, and a high species richness of forbs, especially those of the family Asteraceae (e.g. *Helychrysum* spp., *Senecio* spp.) (Mucina and Rutherford 2006). Although this vegetation unit is generally not regarded as highly threatened, in the area of Hogsback, which consitutes the only known habitat of *Apterotoxitiades vivesi*, most of it has already experienced large-scale transformation to pine plantations (Figure 6).

The generally steep slopes of the Northern Drakensberg Highland Grassland support short sour grassland rich in forbs. Scattered trees of *Protea caffra* and *Protea roupelliae* are also a typical feature of this vegetation unit, as are small patches of mistbelt forest occasionally growing in wet ravines. Unlike the previous unit, the Northern Drakensberg Highland Grassland vegetation currently faces little conservation threat, particularly in the relatively large uKhahlamba Drakensberg Park, which enjoys status of UNESCO World Heritage Site since 2000 (Mucina and Rutherford 2006). The Royal Natal National Park, where the holotype of *Apterotoxitiades aspinosus* sp. n. was collected in 1972 (Figure 7), currently falls within this wider Park.

All specimens of *Apterotoxitiades vivesi*, including the holotype male collected in August 1992 (Adlbauer 2008), have so far been found under 1–2 year old pine logs lying on the ground, adjacent to the grassland (Figure 6). Three pine species are cultivated in the area by the Amathole Forestry Company, including *Pinus elliottii*, *Pinus patula* and *Pinus radiata* (Chapman 2011). However, no evidence of *Apterotoxitiades vivesi* using this pine wood as boring or larval development medium could be found. All adult specimens were simply hiding under the logs, at the interface between bark and grass or leaf litter, where moisture levels were significantly higher than elsewhere and no light penetration occurred. It is likely that larval development may occur either on the stems of the short shrubs that occur within the grassland, or on the roots of the grass itself. The larvae of some Palearctic genera of Cerambycidae, such as *Vesperus* and *Dorcadion* for instance, are well known for their underground development, feeding on the roots of a variety of grasses and shrubs (Pesarini and Sabbadini 1994).

Given their extremely reduced compound eyes, adult *Apterotoxitiades vivesi* are probably nocturnal in activity. During the period of their activity, this area does not receive any major rainfall, but some surface moisture is maintained by night-time mist/fog. As this however dries out in the heat of the day, the beetles would need to return underground or find a suitable shelter for the day at the surface, ideally rich in moisture and protected from light and visual predators. Thus, tree logs lying on the ground at the edge of the grassland, and possibly also large stones, may provide an ideal hideout for adults to spend the day. However, this habitat is also shared by ground beetles (Carabidae) and spiders, with the latter actually consuming *Apterotoxitiades vivesi*, judging by the state of the carcasses retrieved in their silk wrapping.

The following observations were made directly in the field by R.P. during the survey of September 2014. Remarkably, the only two specimens found still alive in their habitat (all the other specimens were already dead and partly decomposed) died very rapidly once removed from their wet and dark hideout under the wood. They immediately entered a state of muscular spasm, developing a shivering-type of reaction followed by the folding of their legs and death within a period of < 1 hour. This reaction could possibly have been caused by sudden exposure to intense light, as their compound eyes are extremely reduced (Figures 1, 3, 5) and reminiscent of those observed in some cave beetles. A more likely possibility is, however, that they may have suffered thermal shock, by being suddenly exposed to temperatures much higher than those prevailing under the logs. The air temperature on the day of the collection was in fact partricularly high in comparison to seasonal averages, with almost 30 °C attained around midday.

Even more intriguing appears to be the period of adult activity: mid-late winter. The already dead specimens and a few more consumed carcasses found on site, clearly indicate that adults were already on their way out in early September and probably at peak activity about a month earlier. This is unusual for high altitude areas of southern Africa, where adult cerambycids generally start emerging only in the spring, after substantial rainfall events. At Hogsback, in particular, rainfall exhibits a bimodal pattern, with spring and late summer peaks, and annual precipitation can reach 1000 mm. Minimum temperatures often plummet below zero in winter and frost occurs with frequency of up to 80 days per year (Leroux 1994, Mucina and Rutherford 2006). Occasional, light snowfalls are also a regular feature of the winter season. Thus, the winter activity of *Apterotoxitiades vivesi*, combined with its apparent intolerance for high temperatures, may be indicative of an unusual adaptation to cold climatic conditions.

Unfortunately, no habitat data was reported on the label accompanyng the holotype specimen of *Apterotoxitiades aspinosus* sp. n., and thus it is not possible to draw conclusions about its ecology. Nevertheless, it seems likely that its main traits may be similar to those observed in *Apterotoxitiades vivesi*, with the exception that in this case the period of adult activity is clearly in the spring, as the fresh holotype specimen was found in October.

## Supplementary Material

XML Treatment for Apterotoxitiades vivesi

XML Treatment for Apterotoxitiades aspinosus
